# Birth Defects Data From Population-Based Birth Defects Surveillance System in a District of Southern Jiangsu, China, 2014–2018

**DOI:** 10.3389/fpubh.2020.00378

**Published:** 2020-08-06

**Authors:** Ying Zhou, Xueqin Mao, Hua Zhou, Li Wang, Zhiqiang Qin, Zhengmao Cai, Bin Yu

**Affiliations:** ^1^Changzhou Maternity and Child Health Care Hospital Affiliated to Nanjing Medical University, Changzhou, China; ^2^Changzhou Commission of Health, Changzhou, China

**Keywords:** birth defects, prevalence, risk factors, population-based, surveillance

## Abstract

As a population-based national surveillance region, Tianning District confronts with great challenges in birth defects (BDs) prevention. We aimed to describe the epidemiology of BDs in infants (including dead fetus, stillbirth, or live birth between 28 weeks of gestation and 42 days after birth) in Tianning District from 2014 to 2018. The data was collected from the national birth defect surveillance system. The prevalence rates of BDs were calculated by poisson distribution. Trends of incidence and the associations of regarding perinatal characteristics with BDs were analyzed by poisson regression. During the study period, the prevalence of BD was 155.49 per 10,000 infants. The ten leading BDs were congenital heart defects (CHD), polydactyly, Congenital malformation of kidney (CMK), syndactyly, cleft palate, hypospadias, Congenital hypothyroidism (CH), congenital atresia of rectum and anus, congenital talipes equinovarus (CTE), and microtia. A significant increase in the prevalence of CHD was observed with a prevalence rate ratio (PRR) of 1.191. Gravidity ≥ 3 (PRR = 1.38) and multiple births (PRR = 2.88) were risk factors for BDs. Premature delivery (PRR = 4.29), fetal death or stillbirth (PRR = 24.79), and infant death (PRR = 43.19) were adverse consequences of BDs. Strengthening publicity and education, improving the ability of diagnosis and monitoring, expanding surveillance time quantum of BDs system may be warranted.

## Introduction

Birth defects (BDs) or congenital anomalies are defined as a series of structural, functional, or metabolic disorders during the development of the embryo or fetus (1). BDs have caused early miscarriage, fetal death, infant death, childhood disabilities, and have been a global public health issue (2). It was estimated that the incidence rate of BDs in low-income, developing, and developed countries were 64.2, 55.7, and 47.2 per 1,000 live births, respectively (3). In China, 5.6% of total newborns were born with BDs annually, about the level of developing countries (4). The prevalence rates of different BDs have changed immensely in the last few decades (4). Specially, the incidence rates of limb reduction and neural tube defect (NTD) declined tremendously (5), while the prevalence rate of CHD was found to show an increasing trend globally (6). Most BDs arise from a complex and ill-defined combination of genetic and environmental factors (7). There are palpable regional differences in the prevalence of BDs for the fact that risk factor varies widely with time and place. In order to identify the incidence, trends and risk factors of BDs, it is crucial to study their epidemiology.

There are two types of national surveillance systems in China, namely, hospital-based surveillance system (including early fetuses <28 weeks of gestation and perinatal infants between 28 weeks of gestation and 7 days after birth born in hospitals) and population-based surveillance system (including dead fetus, stillbirth, terminations of pregnancy, and live birth between 28 weeks of gestation and 42 days postpartum birth lived in particular districts or counties). It is incomparable to the data in these two systems for different coverage of surveillance time quantum. Several articles reported the epidemiology of BDs in hospital-based surveillance system, but few described the prevalence of BDs in population-based surveillance system. Therefore, reporting the population-based prevalence of BDs is also necessary.

Tianning District is located in the economically developed Taihu Plain of the Yangtze River Delta. Its traffic is convenient and it attracted many migrants. It has diversified industries, with textile-clothing and machanical-electronic as the pillars. Industrial environmental risk factors such as noise have brought challenges to the prevention of adverse pregnancy outcomes (8). Tianning District has become one of the population-based surveillance spots of BDs in China since 2006. In 2018, the monitoring data showed that the prevalence of BDs increased remarkably. In this study, we aimed to describe the epidemiology and characteristics of BDs in Tianning District between 2014 and 2018.

## Methods

### Study Population

The study population included live birth, stillbirth, dead fetus, and legal termination of pregnancy between 28 weeks gestation and 42 days postpartum whose mother lived in Tianning District between 2014 and 2018. The study has complied with the World Medical Association Declaration of Helsinki regarding ethical conduct of research and was approved by the Ethics Committee of Changzhou Maternity and Child Health Care Hospital affiliated to Nanjing Medical University. Participants consented were achieved when community doctors visited parturients 42 days after their delivery.

### Data Collection and Quality Control

According to “Maternal and Child Health Monitoring Manual in China” formulated by the National Office for Maternal and Child Health Surveillance of China, diseases numbered Q00.000-Q99.999 or O35.000-35.206 in the International Classification of Diseases Clinical Modification Codes, 10th revision (ICD-10) were noted as birth defects should be monitored in our surveillance system (4). External birth defects were diagnosed with physical examination, visceral anomalies were mainly diagnosed with ultrasonography or ray examination, and chromosomal or genetic diseases were diagnosed with molecular diagnostic methods. The criteria of BDs diagnosis has been stable since 2003 according to “Maternal and Child Health Monitoring Manual in China.” Professional and trained obstetric or pediatric health care doctors from Community Health Service Centers of Tianning District collected information from parents of birth defect cases and hospitals, and filled the “Birth Defects Registration Form” of every case and “Follow-up Registration Form” of each perinatal infant. Obstetric health care doctors of Tianning Maternal and Child Health Family Planning Service Center input the information to the surveillance system. Information was audited by maternity and child health care hospitals and health administrative departments, respectively. Quality control of the data were conducted quarterly at district-level, half-yearly at city-level, and yearly at province-level or nation-level to ensure the accuracy of relevant information.

### Statistical Analysis

Poisson distribution and poisson regression were performed to calculate the prevalence rates, potential risk factors and adverse consequences of BDs (4). R version 3.5.1 (The Comprehensive R Archive Network: http://cran.r-project.org) was used for data statistical analysis. *P* < 0.05 was considered as representative of statistically significant.

## Results

Annually prevalence rates and trends of total BDs in Tianning District from 2014 to 2018 are summarized in Table 1. From 2014 to 2018, 28,040 infants were registered and 436 BDs were diagnosed in Tianning District, resulting in an incidence of 155.49 (95% CI: 141.24–170.80) per 10,000 infants. Although poisson regression showed no significant increase in the prevalence rates of total BDs during the 5-year study period, the prevalence rates of BDs in 2018 was much higher than those in the other years.

**Table 1:** Prevalence rates and trends of BDs in Tianning District, 2014–2018.

**Year**	BDs^*^(n)	**Infants(n)**	**Prevalences of BDs per 10,000 infants (95% CI)**	**PRR^**#**^(95% CI)**
2014	71	4,671	152.00 (118.71, 191.73)	1.05 (0.98, 1.12)
2015	70	4,608	151.91 (118.42, 191.93)	
2016	96	6,842	140.31 (113.65, 171.34)	
2017	94	6,309	148.99 (120.40, 182.33)	
2018	105	5,610	187.17 (153.08, 226.58)	
Total	436	28,040	155.49 (141.24, 170.80)	/

* *BDs, birth defects*.

# *PRR, prevalence rate ratio*.

As shown in Table 2, during the study period, BDs in circulatory, musculoskeletal, urogenital and digestive systems were more common. Particularly, the 10 most common BDs were CHD, polydactyly, congenital malformation of kidney(CMK), syndactyly, cleft palate, hypospadias, congenital hypothyroidism(CH), congenital atresia of rectum and anus, congenital talipes equinovarus(CTE), and microtia, with incidence rates of 124.11, 19.61, 8.56, 6.42, 4.64, 4.64, 4.28, 4.28, 3.57, and 3.57 per 10,000 infants, respectively. The prevalence rate of CHD (PRR 1.191, 1.099–1.290) increased significantly from 2014 to 2018. Other BDs remained stable through the study period.

**Table 2:** Prevalence rates and trends of selected BDs in Tianning District, 2014–2018 (per 10,000 infants).

Types of BDs^*^	**2014**	**2015**	**2016**	**2017**	**2018**	**Total**	**Rank**	**PRR^**#**^(95% CI)**
**Central nervous system**								
Subependymal cysts	2.14	2.17	0	0	1.78	1.07	14	0.78 (0.34, 1.81)
**Eye, ear, face, and neck**								
Microtia	4.28	4.34	4.38	3.17	1.78	3.57	7	0.84 (0.53, 1.32)
Other malformation of external ear	4.28	4.34	2.92	0	3.57	2.85	9	0.82 (0.49, 1.36)
**Lip, plate**								
Cleft palate	6.42	4.34	5.85	1.59	5.35	4.64	5	0.89 (0.60, 1.33)
Cleft lip	2.14	2.17	5.85	3.17	1.78	3.21	8	0.99 (0.61, 1.60)
Cleft lip and palate	4.28	0	0	1.59	3.57	1.78	12	1.04 (0.54, 1.99)
**Circulatory system**								
Congenital heart defects	107.07	97.66	86.23	142.65	185.38	124.11	1	**1.19 (1.10, 1.29)**^**a**^
**Digestive system**								
Congenital atresia of rectum and anus	4.28	10.85	4.38	0	3.57	4.28	6	0.74 (0.49, 1.14)
Congenital gastric volvulus	2.14	0	1.46	0	3.57	2.50	10	1.41 (0.77, 2.57)
Congenital atresia of intestine	4.28	2.17	0	0	1.78	2.14	11	0.61 (0.28, 1.33)
Congenital pyloristenosis	6.42	2.17	1.46	0	1.78	2.14	11	0.58 (0.30, 1.11)
Gastrointestinal obstruction	2.14	4.34	0	1.59	0	1.43	13	0.61 (0.28, 1.33)
Congenital esophageal atresia	4.28	0	0	1.59	0	1.07	14	0.51 (0.19, 1.37)
**Urogenital system**								
Congenital malformation of kidney	10.70	6.51	14.62	3.17	7.13	8.56	3	0.87 (0.65, 1.17)
Hypospadias	8.56	6.51	2.92	3.17	3.57	4.64	5	0.76 (0.50, 1.14)
Cryptorchidism	0	0	1.46	1.59	1.78	1.07	14	1.73 (0.63, 4.78)
**Musculoskeletal system**								
Polydactyly	19.27	17.36	24.85	17.44	17.83	19.61	2	0.98 (0.81, 1.19)
Syndactyly	6.42	2.17	11.69	7.93	1.78	6.42	4	0.93 (0.67, 1.31)
Congenital talipes equinovarus	4.28	0	2.92	4.76	5.35	3.57	7	1.23 (0.76, 1.99)
Umbilical hernia	0	6.51	1.46	1.59	1.78	2.14	11	1.55 (0.83, 2.91)
Congenital diaphragmatic hernia	0	0	2.92	1.59	0	1.07	14	1.12 (0.48, 2.62)
**Endocrine system**								
Congenital hypothyroidism	2.14	4.34	2.92	6.34	5.35	4.28	6	1.23 (0.80, 1.91)
**Chromosomal abnormalities**								
Down's syndrome	0	2.17	1.46	1.59	0	1.07	14	0.93 (0.41, 2.14)
**Other**								
Hemangioma	0	2.17	2.92	1.59	5.35	2.50	10	1.55 (0.83, 2.91)

* *BDs, birth defects*.

# *PRR, prevalence rate ratio*.

a *The prevalence of CHD increased by 19.00% per year on average during the study period*.

The incidence rates of different infant gender, five maternal age groups, different gravidity and parity as well as results of poisson regression are summarized in Table 3. The prevalence rate of BDs in women with gravidity ≥ 3 was significantly higher than that in women with gravidity = 1 (194.38 vs. 140.51 per 10,000 infants, PRR = 1.38, 95% CI: 1.09–1.75). After adjusting for maternal age, this association still existed (adjusted PRR = 1.52, 95% CI: 1.18–1.96). The incidence rate of BDs in multiple births was significantly higher than that in singleton births (426.36 vs. 147.82 per 10,000 infants, PRR = 2.88, 95% CI: 2.02–4.11). Moreover, as shown in Table 3, infants with BDs were more likely to become premature infants (PRR = 4.29, 95% CI: 3.39–5.44), fetal death or stillbirths (PRR = 24.79, 95% CI: 16.42–37.43), and infant death (PRR = 43.19, 95% CI: 27.56–67.69). However, we did not detect significant differences on prevalence of BDs between infant gender groups or maternal age groups.

**Table 3:** Prevalence rates of BDs analyzed from the perspective perinatal characteristics.

**Characteristics**		BDs^*^(n)	**Infants(n)**	**Prevalences of BDs per 10,000 infants (95% CI)**	**PRR^**#**^(95% CI)**
Infant gender	Male	245	14,603	167.77 (147.42, 190.15)	1.18 (0.98, 1.43)
	Female	191	13,437	142.14 (122.70, 163.80)	Reference
Maternal age	<20	5	327	152.91 (49.65, 356.83)	0.99 (0.41, 2.39)
	20–24	69	3,933	175.44 (136.50, 222.03)	1.13 (0.86, 1.48)
	25–29	209	13,473	155.13 (134.81, 177.64)	Reference
	30~34	110	7,042	156.21 (128.38, 188.27)	1.01 (0.80, 1.27)
	35~	43	3,258	131.98 (95.52, 177.78)	0.85 (0.61, 1.18)
Gravidity	1	187	13,309	140.51 (121.09, 162.15)	Reference
	2	137	8,531	160.59 (134.83, 189.84)	1.14 (0.92, 1.42)
	≥3	112	5,762	194.38 (160.05, 233.89)	**1.38 (1.09, 1.75)**^**a**^
Type of pregnancy	Multiple	33	774	426.36 (293.48, 598.76)	**2.88 (2.02, 4.11)**^**b**^
	Singleton	403	27,262	147.82 (133.74, 162.99)	Reference
Premature delivery	Yes	86	1,518	566.53 (453.15, 699.67)	**4.29 (3.39, 5.44)**^**c**^
	No	350	26,522	131.97 (118.50, 146.54)	Reference
Birth outcome	Stillbirth and fetal death	24	69	3,478.26 (2,228.59, 5,175.38)	**24.79 (16.42, 37.43)**^**d**^
	Infant death	20	33	6,060.61 (3,701.98, 9,360.12)	**43.19 (27.56, 67.69)**^**d**^
	Live birth	392	27,937	140.32 (126.77, 154.92)	Reference

* *BDs, birth defects*.

# *PRR, prevalence rate ratio*.

a *The risk of BDs in infants delivered by women with gravidity ≥ 3 was 1.38 times higher than those delivered by women with gravidity = 1*.

b *The risk of BDs in multiparity was 2.88 times higher than those in single pregnancy*.

c *The risk of BDs in premature infants was 4.29 times higher than those in term infants*.

d *The risk of BDs in still birth and fetal death was 24.79 times higher than those in live birth. The risk of BDs in infant death was 43.19 times higher than those in live birth*.

## Discussion

In the current study, we detected significant increase in the prevalence of CHD with a PRR of 1.191. BDs in circulatory, musculoskeletal, urogenital and digestive systems were more common. Gravidity ≥ 3 and multiple births were risk factors for occurrence of BDs, and premature delivery, fetal death or stillbirth, and infant death were adverse consequences of BDs.

In our study, 24.31 percent (106/436) infants with BDs were found between 8 days and 42 days after their birth. Also, with the advancing of diagnosis skills, many BDs can be found before 28 weeks of gestation and pregnant women with severe BDs might choose the termination of pregnancy (9). Expanding time quantum of surveillance and collecting information of women with severe BDs choosing termination <28 weeks of gestation are necessary to get an exact disease spectrum of BDs so as to make better prevention strategy. Besides, circulatory, musculoskeletal, urogenital and digestive systems were susceptible to BDs and more attention should be paid to them.

The prevalence of CHD was the highest among all kinds of BDs during the study period. Moreover, it varied between 86.23/10,000 and 185.38/10,000. An European study revealed that the incidence rates of CHD had increased 9 times in recent decades (6). The increase in CHD incidence over time is caused by advance in diagnostic means such as better, more normative and accessible perinatal B-mode ultrasonography services (10–12). Also, potential risk factors including environmental pollution such as specific combustion pollutants, occupational hazards, and mental stress could influence the prevalence of CHD (8, 13). As its serious hazards to sick infants and children, much attention should be paid to the prevention and screening of CHD. Avoiding exposure to environmental and occupational hazards during early pregnancy, joyful mood, and regular perinatal examination are effective measures.

We found that CMK and hypospadias were the third and fifth highest among all kinds of BDs, which is a little higher than the rank of Hunan Province (14). Urinary malformations are caused by complex interactions between genetic and environmental factors, some of which have been identified (15). It is suggested that maternal obesity, diabetes, kidney disease, low birth weight, smoking and drinking during pregnancy result in urinary anomalies (16–19). Moreover, other studies have suggested that environmental pollutants such as chlorination disinfection byproduct in drinking water, TCDD (2,3,7,8-tetracholrodibenzo-p-dioxin), endocrine disrupting chemicals (EDCs) are associated with occurrence of CMK or hypospadias (20–22). Therefore, building up a healthy life style, avoiding exposure to environmental pollutants, and paying attention to protection at work are important measures to cut down the prevalence rates of CMK and hypospadias.

Cleft palate was the fifth highest among all types of BDs in our study. It is estimated that cleft palate affecting 1–25 per 10,000 newborns worldwide (23). The potential risk factors for cleft palate have been extensively researched through epidemiologic and experimental studies, which included maternal exposure to tobacco smoke, alcohol, and corticosteroids; folic acid deficiency; zinc deficiency; maternal grief; paternal advanced age; and paternal smoking or alcohol use (24–30). Gene- environment interactions such as potential interaction between *TBX4* (chromosome17q21-q22) and dietary folate might also contribute to the occurrence of cleft palate (31). Therefore, smoking cessation, limited alcohol, balanced nutrition, joyful mood, pregnancy at appropriate age, and improving the technology of prenatal diagnosis are crucial for the prevention of cleft palate.

CH was the sixth highest among all kinds of BDs in our study. CH is a common endocrine disease in newborns and affects ~1 in 2,000–4,000 live births (32). According to the national network of neonatal screening centers, the average prevalence of CH in China was 1 per 2,047 between 1985 and 2007, and the prevalence rate increased significantly during the study period (33). Multiple factors including ethnicity, characteristics of birth and pregnancy, and screening programs associate with the occurrence of CH (34). The most common clinical features of CH were delayed physiological development, constipation, jaundice, and intellectual disability (35). Fortunately, CH could be controlled at steady status by early detection and timely treatment, and neonatal screening is an sensitive measure to detect CH. Popularizing neonatal screening, following up and managing CH cases effectively are necessary to guarantee the life quality of CH cases.

The authors found that women with gravidity ≥3 were more likely to give birth to infants with BDs, and after adjusting for maternal age, this association still remained. Li et al. reported that gravidity was associated with occurrence of CHD (13). In general, the increase of gravidity indicates the increase of accidental pregnancy, unwanted pregnancy, and termination of pregnancy, which were risk factors of high-risk births (35). So it is necessary to take effective contraception measures and provide birth health counseling. Besides, multiple births were risk factors for occurrence of BDs. In order to cut down the birth rate of severe BDs, perinatal examination for multiple births should be more careful. In addition, similar to Kancherla's study, premature delivery, fetal death or stillbirth, and infant death were adverse consequences of BDs (2). However, we did not detect significant association between infant gender and BDs, which is different from Xie's study (14). Although males are more susceptible to BDs for Y chromosome and their external genital malformations are more detectable (36, 37), with the expansion of surveillance time quantum, several genital deformities can be diagnosed in female infants. Moreover, some BDs mostly diagnosed after birth such as cleft palate and CH are more often in female infants (23, 34). Besides, some studies reported that maternal age had association with occurrence of BDs (14, 38), but we did not detect the association in the present study. The probable reasons are as follows: on the one hand, the authors were underpowered to detect differences between maternal age and BDs because of small sample sizes of pregnant women younger than 20 or older than 35; on the other hand, pregnant women younger than 20 tend to choose the termination of pregnancy at small gestational age for not being allowed to get married, so do pregnant women older than 35 for serious BDs such as chromosome aberration (39). Thus, the impact of infant gender and maternal age on BDs needs further research.

## Limitations

There are several limitations in our study. First, different types of BDs have different causes and pathogenesis, it is better to collect information on risk factors including genetic and environmental elements of BDs, and report the association between risk factors and BDs individually. Second, without materials concerning termination at small gestational age in pregnant women with serious BDs could underestimate the incidence rate of BDs. Third, the study is limited by the size of the study population. Pooling data from several national population-based birth defect surveillance regions can get more meaningful results.

## Conclusions

In summary, a significant increase in the prevalence of CHD was detected from 2014 to 2018 in Tianning District. Gravidity ≥ 3 and multiple births were risk factors for occurrence of BDs. Besides, infants with BDs were more likely to become premature infants, fetal death or stillbirths, and infant death. In order to make more targeted prevention strategy, we need to expand surveillance time quantum of BD surveillance system and collect risk factors associated with BDs. Besides, strengthening health education on birth health counseling, regular perinatal examination and improving the skill of diagnosis and monitoring are crucial to lower the incidence of BDs.

## Data Availability Statement

The raw data supporting the conclusions of this article will be made available by the authors, without undue reservation.

## Ethics Statement

The studies involving human participants were reviewed and approved by the Ethics Committee of Changzhou Maternity and Child Health Care Hospital affiliated to Nanjing Medical University. The patients/participants provided their written informed consent to participate in this study.

## Author Contributions

YZ conducted the literature review, statistical analysis, manuscript writing, and critical revision of the manuscript. BY directed the implementation of the study and revised the manuscript. ZC designed and supervised the study. XM, HZ, LW, and ZQ designed the medical part of the study protocol and supervised the field activities (diagonosis of cases, data collection, and quality control). All authors read and approved the final paper.

## Conflict of Interest

The authors declare that the research was conducted in the absence of any commercial or financial relationships that could be construed as a potential conflict of interest.

## References

[1] WHO Primary health care approaches for prevention and control of congenital and genetic disorders. Report of WHO Meeting. (1999). p. 4–8.

[2] Kancherla V Oakley GP Jr Brent RL. Urgent global opportunities to prevent birth defects. Semin Fetal Neonatal Med. (2014) 19:153–60. 10.1016/j.siny.2013.11.008 24333206

[3] The foundation of March of Dimes in American Global Report on Birth Defects. (2006).

[4] Dai L Zhu J Liang J Wang YP Wang H Mao M Birth defects surveillance in China. World J Pediatr. (2011) 7:302–10. 10.1007/s12519-011-0326-0 22015723

[5] Loane M Dolk H Kelly A Teljeur C Greenlees R Densem J. Paper 4: EUROCAT statistical monitoring: identification and investigation of ten year trends of congenital anomalies in Europe. Birth Defects Res A Clin Mol Teratol. (2011) 91(Suppl. 1):S31–43. 10.1002/bdra.20778 21381187

[6] Van der Linde D Konings EE Slager MA Witsenburg M Helbing WA Takkenberg JJ. Birth prevalence of congenital heart disease worldwide: a systematic review and meta-analysis. J Am Coll Cardiol. (2011) 58:2241–7. 10.1016/j.jacc.2011.08.025 22078432

[7] Brent RL. Environmental causes of human congenital malformations: the pediatrician's role in dealing with these complex clinical problems caused by a multiplicity of environmental and genetic factors. Pediatrics. (2004) 113(Suppl 4):957–68. 15060188

[8] Liu X 249 Cases the Incidence of Birth Defects in Children With Risk Factors of Investigation and Analysis. Changchun: Jilin University (2011). p. 1–38.

[9] Leung TN Ching Chau MM Chang JJ Leung TY Fung TY Lau TK. Attitudes towards termination of pregnancy among Hong Kong Chinese women attending prenatal diagnosis counselling clinic. Prenat Diagn. (2004) 24:546–51. 10.1002/pd.950 15300746

[10] Bravo-Valenzuela NJ Peixoto AB Araujo Junior E. Prenatal diagnosis of congenital heart disease: a review of current knowledge. Ind Heart J. (2018) 70:150–64. 10.1016/j.ihj.2017.12.005 29455772 PMC5903017

[11] Alves Rocha L Araujo Júnior E Rolo LC Barros FS Silva KP Martinez LH. Screening of congenital heart disease in the second trimester of pregnancy: current knowledge and new perspectives to the clinical practice. Cardiol Young. (2014) 24:388–96. 10.1017/S1047951113001558 24229491

[12] Plana MN Zamora J Suresh G Fernandez-Pineda L Thangaratinam S Ewer AK. Pulse oximetry screening for critical congenital heart defects. Cochrane Database Syst Rev. (2018) 3:Cd011912. 10.1002/14651858.CD011912.pub2 29494750 PMC6494396

[13] Li H Luo M Zheng J Luo J Zeng R Feng N. An artificial neural network prediction model of congenital heart disease based on risk factors: a hospital-based case-control study. Medicine. (2017) 96:e6090. 10.1097/MD.0000000000006090 28178169 PMC5313026

[14] Xie D Yang T Liu Z Wang H. Epidemiology of birth defects based on a birth defect surveillance system from 2005 to 2014 in hunan province, China. PLoS ONE. (2016) 11:e0147280. 10.1371/journal.pone.0147280 26812057 PMC4728203

[15] Chevalier RL Thornhill BA Forbes MS Kiley SC. Mechanisms of renal injury and progression of renal disease in congenital obstructive nephropathy. Pediatr Nephrol. (2010) 25:687–97. 10.1007/s00467-009-1316-5 19844747

[16] Shnorhavorian M Bittner R Wright JL Schwartz SM. Maternal risk factors for congenital urinary anomalies: results of a population-based case-control study. Urology. (2011) 78:1156–61. 10.1016/j.urology.2011.04.022 22054394

[17] Macumber I Schwartz S Leca N. Maternal obesity is associated with congenital anomalies of the kidney and urinary tract in offspring. Pediatr Nephrol. (2017) 32:635–42. 10.1007/s00467-016-3543-x 27858197

[18] Groen In 't Woud S Renkema KY Schreuder MF Wijers CH van der Zanden LF Knoers NV. Maternal risk factors involved in specific congenital anomalies of the kidney and urinary tract: a case-control study. Birth Defects Res A Clin Mol Teratol. (2016) 106:596–603. 10.1002/bdra.23500 27040999

[19] Luyckx VA Brenner BM. Low birth weight, nephron number, and kidney disease. Kidney Int Suppl. (2005) 68:S68–77. 10.1111/j.1523-1755.2005.09712.x 16014104

[20] Hwang BF Magnus P Jaakkola JJ. Risk of specific birth defects in relation to chlorination and the amount of natural organic matter in the water supply. Am J Epidemiol. (2002) 156:374–82. 10.1093/aje/kwf038 12181108

[21] Abbott BD Birnbaum LS Pratt RM. TCDD-induced hyperplasia of the ureteral epithelium produces hydronephrosis in murine fetuses. Teratology. (1987) 35:329–34. 10.1002/tera.1420350307 3629513

[22] Warembourg C Botton J Lelong N Rouget F Khoshnood B Le Gleau F Prenatal exposure to glycol ethers and cryptorchidism and hypospadias: a nested case-control study. Occup Environ Med. (2018) 75:59–65. 10.1136/oemed-2017-104391 29055888

[23] Burg ML Chai Y Yao CA Magee W 3rd Figueiredo JC. Epidemiology, etiology, and treatment of isolated cleft palate. Front Physiol. (2016) 7:67. 10.3389/fphys.2016.00067 26973535 PMC4771933

[24] Sabbagh HJ Hassan MH Innes NP Elkodary HM Little J Mossey PA. Passive smoking in the etiology of non-syndromic orofacial clefts: a systematic review and meta-analysis. PLoS ONE. (2015) 10:e0116963. 10.1371/journal.pone.0116963 25760440 PMC4356514

[25] Munger RG Tamura T Johnston KE Feldkamp ML Pfister R Carey JC. Plasma zinc concentrations of mothers and the risk of oral clefts in their children in Utah. Birth Defects Res A Clin Mol Teratol. (2009) 85:151–5. 10.1002/bdra.20516 19067407 PMC3712612

[26] Bell JC Raynes-Greenow C Turner RM Bower C Nassar N O'Leary CM. Maternal alcohol consumption during pregnancy and the risk of orofacial clefts in infants: a systematic review and meta-analysis. Paediatr Perinat Epidemiol. (2014) 28:322–32. 10.1111/ppe.12131 24800624

[27] Nakamura A Osonoi T Terauchi Y. Relationship between urinary sodium excretion and pioglitazone-induced edema. J Diabetes Invest. (2010) 1:208–11. 10.1111/j.2040-1124.2010.00046.x 24843434 PMC4020723

[28] Ingstrup KG Liang H Olsen J Nohr EA Bech BH Wu CS. Maternal bereavement in the antenatal period and oral cleft in the offspring. Hum Reprod. (2013) 28:1092–9. 10.1093/humrep/des434 23293222

[29] Herkrath AP Herkrath FJ Rebelo MA Vettore MV. Parental age as a risk factor for non-syndromic oral clefts: a meta-analysis. J Dentistr. (2012) 40:3–14. 10.1016/j.jdent.2011.10.002 22019990

[30] Krapels IP Zielhuis GA Vroom F de Jong-van den Berg LT Kuijpers-Jagtman AM van der Molen AB. Periconceptional health and lifestyle factors of both parents affect the risk of live-born children with orofacial clefts. Birth Defects Res A Clin Mol Teratol. (2006) 76:613–20. 10.1002/bdra.20285 16955502

[31] Skare O Jugessur A Lie RT Wilcox AJ Murray JC Lunde A. Application of a novel hybrid study design to explore gene-environment interactions in orofacial clefts. Ann Hum Genet. (2012) 76:221–36. 10.1111/j.1469-1809.2012.00707.x 22497478 PMC3334353

[32] Hinton CF Harris KB Borgfeld L Drummond-Borg M Eaton R Lorey F. Trends in incidence rates of congenital hypothyroidism related to select demographic factors: data from the United States, California, Massachusetts, New York, and Texas. Pediatrics. (2010) 125(Suppl. 2):S37–47. 10.1542/peds.2009-1975D 20435716

[33] Zhan JY Qin YF Zhao ZY. Neonatal screening for congenital hypothyroidism and phenylketonuria in China. World J Pediatr. (2009) 5:136–9. 10.1007/s12519-009-0027-0 19718537

[34] Alimohamadi Y Taghdir M Sepandi M. Statistical data analysis of the risk factors of neonatal congenital hypothyroidism in khuzestan province, Iran. Data Brief. (2018) 21:2510–4. 10.1016/j.dib.2018.11.113 30560160 PMC6288456

[35] Dorreh F Chaijan PY Javaheri J Zeinalzadeh AH. Epidemiology of congenital hypothyroidism in markazi province, Iran. J Clin Res Pediatr Endocrinol. (2014) 6:105–10. 10.4274/jcrpe.1287 24932604 PMC4141571

[36] Deng X Berletch JB Nguyen DK Disteche CM. X chromosome regulation: diverse patterns in development, tissues and disease. Nat Rev Genet. (2014) 15:367–78. 10.1038/nrg3687 24733023 PMC4117651

[37] Sokal R Tata LJ Fleming KM. Sex prevalence of major congenital anomalies in the United Kingdom: a national population-based study and international comparison meta-analysis. Birth Defects Res A Clin Mol Teratol. (2014) 100:79–91. 10.1002/bdra.23218 24523198 PMC4016755

[38] Luo YL Cheng YL Gao XH Tan SQ Li JM Wang W. Maternal age, parity and isolated birth defects: a population-based case-control study in Shenzhen, China. PLoS ONE. (2013) 8:e81369. 10.1371/journal.pone.0081369 24282587 PMC3839887

[39] Rousseau T Ferdynus C Thauvin-Robinet C Gouyon JB Sagot P. Impact of maternal age distribution on the expected live birth prevalence of down's syndrome in the metropolitan France between 1965 and 2008. J Gynecol Obstet Biol Reprod. (2010) 39:284–9. 10.1016/j.jgyn.2010.03.001 20381272

